# Motion Plan of Maritime Autonomous Surface Ships by Dynamic Programming for Collision Avoidance and Speed Optimization

**DOI:** 10.3390/s19020434

**Published:** 2019-01-21

**Authors:** Xiongfei Geng, Yongcai Wang, Ping Wang, Baochen Zhang

**Affiliations:** 1 School of Software and Microelectronics, Peking University, Beijing 100871, China; 1401110618@pku.edu.cn; 2 China Waterborne Transport Research Institute, Beijing 100088, China; zbc@wti.ac.cn; 3 Department of Computer Sciences, Renmin University of China, Beijing 100872, China

**Keywords:** motion plan, speed optimization, unmanned surface vehicle, velocity obstacle, conventional ships, dynamic programming, maritime autonomous surface ships

## Abstract

Maritime Autonomous Surface Ships (MASS) with advanced guidance, navigation, and control capabilities have attracted great attention in recent years. Sailing safely and efficiently are critical requirements for autonomous control of MASS. The MASS utilizes the information collected by the radar, camera, and Autonomous Identification System (AIS) with which it is equipped. This paper investigates the problem of optimal motion planning for MASS, so it can accomplish its sailing task early and safely when it sails together with other conventional ships. We develop velocity obstacle models for both dynamic and static obstacles to represent the potential conflict-free region with other objects. A greedy interval-based motion-planning algorithm is proposed based on the Velocity Obstacle (VO) model, and we show that the greedy approach may fail to avoid collisions in the successive intervals. A way-blocking metric is proposed to evaluate the risk of collision to improve the greedy algorithm. Then, by assuming constant velocities of the surrounding ships, a novel Dynamic Programming (DP) method is proposed to generate the optimal multiple interval motion plan for MASS. These proposed algorithms are verified by extensive simulations, which show that the DP algorithm provides the lowest collision rate overall and better sailing efficiency than the greedy approaches.

## 1. Introduction

In recent years, Maritime Autonomous Surface Ships (MASS), which have the capabilities of smart sensing, advanced guidance, and autonomous control, have been quickly developed. The advantage of MASS is that they utilize sophisticated sensing, artificial intelligence, and machine learning methods to enable autonomous navigation, which is revolutionizing future maritime transportation [1,2]. The aim of developing MASS is to relax the toil and pain of sailors and captains, to accomplish autonomous sailing even in hazardous ocean environments. However, currently, sailing safety and efficiency are still critical problems for autonomous control of the MASS. These have attracted great research attention.

An MASS is generally equipped with radar, LiDAR, and camera sensors [3], which can detect surrounding mobile objects in real time. It obtains its real-time location and relative location with respect to the shore-side from GPS (Global Positioning System), AIS (Autonomous Identification System) [4], and GIS (Geographical Information Systems) [5]. Based on this information, autonomous motion planning and control are performed by an on-line control and decision system. The autonomous control is a critical problem for an MASS to sail in the dynamic maritime environment. There are two phases of motion planing for an MASS:

1. Long-term path planning [6], which considers the optimization of the sailing route or path selection. This long-term route planning is mainly conducted off-line by the ship company, based on the information of the waterway routes and the sailing task information.
2. Short-term motion planning [7], which considers the short-term moving speed and moving direction planning to avoid collisions and to optimize sailing performances. The short-term path planning is mainly conducted by the autonomous ship or by a remote control center in real time based on the on-site situation detected by the MASS’s on-board sensing system.

The algorithms and methods of long-term motion planning include shortest path algorithms [8] or routing algorithms on graphs [9]. For short-term motion planning, great research attention has been devoted to the investigation of collision avoidance and the navigation of autonomous cars and mobile robots [10,11]. In this paper, the related works are provided in Section 2, and we focus on the motion planning problem of MASS. There are some differences in short-term motion planning of MASS compared to that of Unmanned Ground Vehicles (UGV).

1. First, the size of MASS is larger, and the hydrodynamic force and the inertia force undertaken by MASS are larger than those of UGVs. The MASS motion cannot respond to the control as agilely as UGVs. This requires the short-term motion planning of MASS to be more predictive to take the latency caused by the inertia and the hydrodynamic forces into consideration.
2. Secondly, AIS (Autonomous Identification System), which provides the location, velocity, size, and type of surrounding ships, has been widely installed on today’s vessels. Therefore, the MASS can obtain more information about surrounding agents than UGVs.
3. Thirdly, there is no explicit lane constraint in the waterway; the MASS only needs to guarantee its sailing safety against the shore-side and the other ships. The traffic is also less crowded in waterways than that on roads. As a consequence, the MASS has a larger solution space for sailing speed and direction selection.
4. Fourthly, the MASS must avoid overturning in the water, which requires the MASS to sail smoothly.

Due to these differences, an MASS can obtain better surrounding information including information about remote ships. The motion plan has a larger decision space, but needs to be more predictive to tolerate inertia to maintain sailing smoothness. The MASS can conduct both the short-term motion plan and multiple interval motion plan online with more considerations of future impacts, because of the knowledge of remote ships. This paper investigates the case when an MASS is sailing in a waterway when there are other MASS and conventional vessels present, which will be a general scenario of MASS application in the near future. We consider the following motion control problem:

**Problem** **1.** *Suppose the waterway map is given, which is denoted by M, indicating the feasible area where the MASS can sail. The MASS starts from S∈M, and the destination of the MASS is given as T∈M. We consider a group of conventional ships, and other MASS are sailing in the same map M at their own will. The considered MASS plans its velocity and direction (denoted by a vector $V_{t}$) in a time-interval manner to optimize its motion. The interval length is denoted by $\Delta_{t}$. The goal of the MASS is to reach the target as early as possible, while maintaining sailing safety and smoothness.*

We assume the MASS can obtain surrounding ship information in real time, including the position $\left\{Y_{i},i=1,\cdots ,N\right\}$, velocity $\left\{V_{i},i=1,\cdots ,N\right\}$, and identification of surrounding ships $\left\{C_{i},i=1,\cdots ,N\right\}$ via radar, AIS, and camera sensing systems. Based on this information, the MASS adjusts its motion plan in every successive time interval by adjusting $V_{t}$ to $V_{t+1}=V_{t}+A_{t}$, where $A_{t}$ is the acceleration vector at time *t*. In each interval, the MASS can only adjust its velocity under the constraint of acceleration limits and turning limitations. The MASS needs also to make sure it is free from collisions with surrounding ships and the map obstacles. Although the short-term motion planning problem has been widely studied for robots and UGVs [8,9,10,11,12], the problem of using the neighborhood extentand remote information to approach optimal motion control by dynamic programming has not been reported in existing works. The main contributions of this paper are as follows:

1. We exploit the Velocity Obstacle (VO) model to represent the collision avoidance constraints with surrounding ships. The VO model for multiple mobile and static objects is presented.
2. A multiple objective optimization problem is formulated for the short-term motion planning problem. The motion smoothness, risk of collision, and sailing efficiency are jointly optimized.
3. A greedy interval-based motion control method is proposed, but we show that the greedy algorithm may fail to avoid collisions in future intervals. We therefore present a way-blocking metric to evaluate the future collision risks, which enables a greedy-risk interval-based motion-planning algorithm.
4. Then, by assuming the other ships are sailing at a constant velocity, the VOs in future time intervals can be calculated by the given state of the MASS. A dynamic programming-based method to seek the optimal motion trajectory is proposed for the sailing safety, efficiency, and smoothness.
5. Extensive simulations are conducted to show the performances of the greedy, greedy-risk, and DP methods. It is shown that the DP method has better performance than the other two methods in both collision avoidance and sailing efficiency.

The remaining sections of the paper are organized as follows. Related works are presented in Section 2. The VO model is presented in Section 3. The greedy and greedy-risk algorithms are presented in Section 4. The multiple interval dynamic programming-based motion optimization method is presented in Section 5. The simulation results are presented in Section 6. The paper is concluded in Section 7 with remarks.

## 2. Related Work

Motion planning of autonomous vehicles for collision avoidance and moving efficiency has attracted great attention. Related works can be roughly categorized into path-planning algorithms and short-term motion planning algorithms.

### 2.1. Path Planning

Early algorithms firstly investigated the problem to ensure the robot moved to the goal in very short time. $A^{*}$ search [8] is a well-known best-first graph search algorithm, which is based on a static environment. $D^{*}$ Search and focused $D^{*}$ [9] can be used for dynamic path planning. They conduct path replanning when dynamic events happens. Field $D^{*}$ [13] is the extension of $D^{*}$ and $D^{*}$ Lite, which uses linear interpolation in order to generate paths with low-cost. Geng et al. [14] proposed the lane-based optimal routing protocol for delay-tolerant maritime networks, which plans the ship path based on predicted meeting times.

Later path planning algorithms focused on mobile robots’ navigation in complex environments. The path-planning algorithm using corridor maps [15] was developed to enhance the smooth trajectory of the robot moving from the start point to the target point. It uses a corridor map and one force to restrict the motion to collision-free paths. The Bugalgorithm [16] has three versions, i.e., Bug-1 algorithm, Bug-2 algorithm, and Distance-Bug algorithm. They all contain a move-to-goal mode and a collision avoidance mode. In collision avoidance mode, the robot moves along the side of the obstacle while planning a new move-to-goal path. The Follow the Gap Method (FGM) [17] finds the widest gap among the obstacles and navigates the robot to move through the center of the obstacles. Path planning using particle swarm optimization [18] tries to speed up the path planning decision. Recent work used the Convolutional Neural Network (CNN) [19] to tackle the path planning problem for visual-based robots. The path planning problem is converted into an environment classification problem based on visual information.

### 2.2. Motion Planning

Different from path planning, motion planning considers the short-term velocity control of the robot more. The robot uses on-board sensors to observe the nearest fraction of an unknown environment for iterative re-computation of a short-horizon trajectory. Many of the related techniques, such as the dynamic window [20] the curvature velocity [10], and the lane curvature [21] approaches treat the obstacles as static. On the other side, approaches like velocity obstacles [12,22,23,24] or collision cones [25] assume a deterministic knowledge about the obstacle velocity and a moderate rate of its change.

(1) Environment model: Exiting methods use different methods to model the environments. Occupancy Grid Map (OGM) [26], VO [12,22,23,24], and state-time space [27] are representative methods. The occupancy grid method represents the environment with grids. The presence of mobile obstacles is represented by grid occupancy. The mobile objects can be represented by the dynamic change of the occupancy grid map. On the other hand, the VO model represents a moving object with nearly constant velocity by a velocity vector. The geometric relationship between the robot and the surrounding mobile objects can be inferred by the relationships among the velocity vectors. The feasible motion vectors of the robot can therefore be inferred from the geometric relationship. State-time space is a tool to formulate trajectory planning in dynamic workspace problems. It permits us to study the different aspects of dynamic trajectory planning, i.e., moving obstacles and dynamic constraints, in a unified way. A near-time-optimal approach that searches the solution trajectory over a restricted set of canonical trajectories was presented in [27]. Using the state-time space model, it is possible to transform the problem of finding the time-optimal canonical trajectory to finding the shortest path in a directed graph embedded in the state-time space.

(2) Motion control algorithms: Based on the environment model, motion control algorithms have been widely investigated. Model Predictive Control (MPC) [28] is the one that has been increasingly applied to vehicle navigation. It performs the path planning process at every time instant, then applies the initial control related to the chosen trajectory to the vehicle. MPC has a set of variants, such as min-max MPC, which conducts predictive control under min-max constraints, and nonlinear MPC [29], which uses a nonlinear trajectory tracking system to ensure that the actual state converges to the nominal state. Obstacle avoidance via boundary following is a standard method employed by many obstacle-avoidance algorithms. In this method, the robot moves towards the target location until a threat of collision with an obstacle is detected. Then, to avoid the collision, the robot bypasses the obstacle by following its boundary while temporarily putting aside the main objective. After passing the obstacle, the robot switches back to the main moving objective. In the case that the obstacle information is fully known, including the location, velocity, and shapes, the VisBugclass of algorithms [30] has been proposed, which navigate toward a visible edge of an obstacle inside the detection range.

However, existing motion planning algorithms generally have not taken the motion smoothness and risk of ship overturn into consideration, since most of the existing works are designed for underground robots or UGVs. In addition, the remote information collected by AIS enables the MASS to conduct the on-line motion plan with consideration of far away ships. This enables the dynamic programming approach to the online motion planning, which has been rarely reported in existing studies.

## 3. Velocity Obstacle Model for MASS

In this paper, Problem 1, i.e., the short-term motion control problem for MASS, is investigated. The MASS is assumed to be equipped with radar, LiDAR, GPS, and AIS, so that it can detect information about surrounding ships and obstacles within its sensing radius R on the map M. For the large inertia of the MASS itself and the other ships, the surrounding ships and obstacles can no longer be treated as static obstacles as in most of the robot motion planning works [10,20]. The relative velocity between the MASS and surrounding objects must be considered to avoid collision. We therefore propose to exploit the VO model [12] to investigate the condition of collision avoidance. The shape information of the detected objects can be extracted from the radar scan and ship information collected by the AIS system. We introduce how to build the VO model for surrounding objects for an MASS in this section.

### 3.1. Velocity Obstacle

The VO model was firstly proposed by Fiorini et al. [12] to describe how surrounding mobile objects may impact a mobile robot with respect to collision avoidance. The VO model is based on linear approximation of the object velocities at a time instant. We extend the VO model to the detection of multiple mobile objects and also for the shore-sides and islands on the map.

#### 3.1.1. Relative Velocity Model

**Figure 1:**
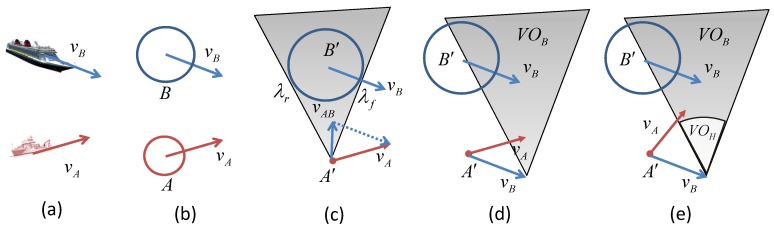
Illustration of the Velocity Obstacle (VO) model. (**a**) Ship *A* is a Maritime Autonomous Surface Ship (MASS), and ship *B* is a conventional ship, which are moving in velocity vector $v_{A}$ and $v_{B}$, respectively; (**b**) represents the geometric shapes of the two ships by circles; (**c**) represents the collision region by relative velocity: if the relative velocity vector $v_{AB}$ falls into the shaded area, the MASS *A* may collide with the ship *B*; (**d**) represents the collision region by the absolute velocity vector of *A*, i.e., if the velocity vector $v_{A}$ ends in the shaded area, the MASS *A* may collide with *B*; (**e**) the velocity obstacle $VO_{B}$ for a short time interval.

Figure 1 illustrates the concepts of the VO model. At a time instant *t*, suppose there is an MASS, denoted by *A*, whose velocity vector is represented by $v_{A}$. There is another ship *B* whose velocity vector is $v_{B}$. Since the shapes of *A* and *B* cannot be known exactly, the VO model firstly abstracts the shape of *A* and *B* by circles of radius $r_{A}$ and $r_{B}$, respectively ($r_{A}$ and $r_{B}$ may be determined by the sizes of the ships). The velocity vectors are then originated at the centers of the circles. In Figure 1c, the problem is transformed to the configuration space of *A* by considering the relative motion between *A* and *B*. The MASS *A* is converted to a point $A^{\prime }$, and the circle *B* is expanded to a larger circle $B^{\prime }$ whose radius is $r_{A}+r_{B}$. The relative speed vector between *A* and *B* is calculated as: $v_{AB}=v_{A}-v_{B}$.

**Definition** **1** (Collision cone of mobile ships)**.** *A collision cone of mobile ship $CC_{AB}$ is defined as a cone-shaped region, centered at $A^{\prime }$ and bounded by the two tangent lines $\lambda_{r}$ and $\lambda_{f}$ from $A^{\prime }$ to $B^{\prime }$, as shown in Figure 1c.*

(1) $$CC{}_{A,B}=\left\{v_{AB}|\lambda_{AB}\cap B^{\prime }\neq \emptyset \right\}$$

*where $\lambda_{l}$ and $\lambda_{r}$ are tangent lines from *A* to $B^{\prime }$ as shown in Figure 1c; $\lambda_{AB}$ is the line extended from the relative velocity vector $v_{AB}$. When the line of the relative velocity vector, i.e., $\lambda_{AB}$ touches $B^{\prime }$, this means the MASS *A* may collide with *B* after some time if their relative velocity is $v_{AB}$. The selections of $V_{AB}$ outside $CC_{AB}$ are safe for collision avoidance.*

If the detected object is a static object on the map, such as the shore-side and islands, the shapes of these objects are generally irregular. However, we can still extend the VO model by considering the relative velocity vector between the MASS and the static object. Since the object is static, the relative velocity $v_{AB}=v_{A}$.

**Figure 2:**
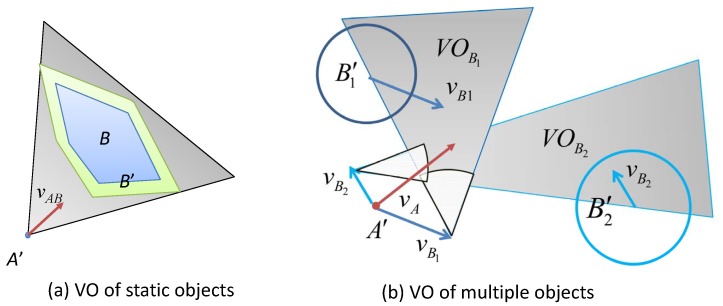
(**a**) VO of static objects; (**b**) illustration of the VO model of multiple mobile objects. $B_{1}$ and $B_{2}$ are two objects. The shaded areas show $VO_{B_{1}}$ and $VO_{B_{2}}$, respectively.

**Definition** **2** (Collision cone of static objects)**.** *A collision cone between an MASS and a static object is defined as $CC_{AB}$, which is a cone-shaped region, centered at $A^{\prime }$ and bounded by the two tangent lines $\lambda_{r}$ and $\lambda_{f}$ from $A^{\prime }$ to $B^{\prime }$ as shown in Figure 2a.*

(2) $$CC_{A,B}=\left\{v_{AB}|\lambda_{AB}\cap B^{\prime }\neq \emptyset \right\}$$

*where $\lambda_{AB}$ is the line extended from the relative velocity vector $v_{AB}$.*

#### 3.1.2. Absolute Velocity Model

In order to determine the feasible space for $v_{A}$, Figure 1d shows the velocity model for the MASS *A*. By adding all vectors in $CC_{A,B}$ with a vector $v_{B}$, we will obtain a cone-shaped region $VO_{B}$. The ⊕ operation in (3) is called the Minkowski vector sum.

(3) $$VO_{B}=CC_{A,B}⊕v_{B}$$

If the velocity vector $v_{A}$ is in $VO_{B}$, the ship *A* may collide with *B* after some time. Therefore, the vectors outside $VO_{B}$ can be selected for $v_{A}$ while keeping collision avoidance with *B*.

#### 3.1.3. VO for a Short Time Interval

Since the control of MASS can be updated in each time interval, we need not consider the potential collisions after a long time. Therefore, only potential collisions within a short time interval should be considered. Therefore, the VO is modified by subtracting the potential collisions that may happen beyond a time interval $T_{H}$.

(4) $$VO_{H,B}=\left\{v_{A}|v_{A}\in VO,∥v_{A,B}∥\leq \frac{d_{A,B}}{T_{H}}\right\}$$

$d_{A,B}$ is the shortest distance between *A* and *B*, and $T_{H}$ is a time interval threshold. Then, we update $VO_{B}=VO_{B}\setminus VO_{H,B}$. The shaded area in Figure 1e shows the velocity model that considers potential collisions within time interval $T_{H}$.

#### 3.1.4. VO for Multiple Mobile Objects

When multiple objects (including mobile and static) are detected surrounding the MASS, the VO model can be calculated by the overlapping area of VOs of all surrounding objects. Suppose there are *k* objects detected in the sensing range of an MASS *A*, whose VOs are denoted by $\left\{VO_{B_{1}},\cdots ,VO_{B_{k}}\right\}$, respectively. Then, the joint VO for multiple objects is:

(5) $$VO_{\left[B_{1}:B_{k}\right]}=VO_{B_{1}}\cup VO_{B_{2}}\cup \cdots \cup VO_{B_{k}}$$

Figure 2b illustrates an example when there are two objects. $B_{1}$ and $B_{2}$ are two objects. The shaded areas show $VO_{B_{1}}$ and $VO_{B_{2}}$, respectively. Only when the velocity vector $v_{A}$ is outside both $VO_{B_{1}}$ and $VO_{B_{2}}$, collision avoidance from $B_{1}$ and $B_{2}$ can be guaranteed.

### 3.2. Building the VO Model

An MASS is generally equipped with different kinds of sensors, including camera, laser radar, and ultrasound radar. The MASS can obtain its own location from GPS and the locations of surrounding ships from AIS. The radar system also detects static objects. Based on this information, an MASS can generate the VO model of surrounding objects in its own configuration space.

Suppose the location of the MASS is at $X_{t}$ and with velocity vector $v_{t}$. There are *K* objects detected, which are denoted by $\left\{B_{1},\cdots ,B_{k}\right\}$. The contours of these objects can be detected by the radar system installed on the MASS. We denote the contours of these objects by $\left\{Y_{1},Y_{2},\cdots ,Y_{K}\right\}$. The velocity vectors of the surrounding objects (including static ones) are denoted by $\left\{v_{B_{1}},v_{B_{2}},\cdots ,v_{B_{K}}\right\}$. Based on this information, the extended contours of surrounding objects can be calculated by shrinking the original contours by a radius $r_{A}$, which is the radius of the MASS. Using this information, the VO model for the MASS at a time instant *t* can be built by Algorithm 1. The algorithm processes each mobile object once and calculates VO for time *t* by the union VOs of all the surrounding objects.

**Algorithm 1:** Building the VO model from sensor readings.

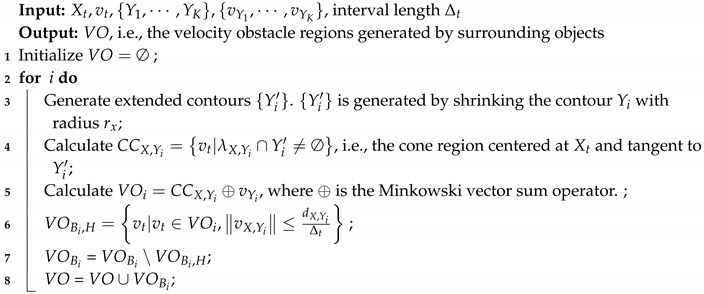



## 4. Multiple Objective Motion Optimization Problem

Based on the VO model built for the MASS at time *t*, the MASS adjusts its velocity to insure not only collision avoidance, but also to optimize the motion pattern to retain motion smoothness and to approach the target efficiently. We assume the control is carried out by adding an acceleration vector to the engine to adjust the velocity during interval $\Delta_{t}$. Since the time interval $\Delta_{t}$ is short, we consider that the acceleration during $\Delta_{t}$ is invariant. If different accelerations are applied, it can be considered that the different accelerations are applied in different intervals. The length of $\Delta_{t}$ can be adjusted for different control resolutions. Based on this, the MASS location and velocity at time $t+\Delta_{t}$ will be:

(6) $$\begin{array}{l} X_{t+1}=X_{t}+V_{t}\Delta_{t}+\frac{A_{t}\cdot \Delta_{t}^{2}}{2}\\ V_{t+1}=V_{t}+A_{t}\Delta_{t} \end{array}$$

Problem 1 is then converted to optimizing the selection of $A_{t}$, at each time *t*, so as to optimize the sailing efficiency, smoothness, and safety.

### 4.1. Motion Optimization Metrics

Three metrics need to be considered for the motion optimization at time interval *t*, including safety, sailing efficiency, and sailing smoothness.

#### 4.1.1. Safety

Safety is the most critical requirement. The MASS must avoid collisions with surrounding objects. Based on the VO model, this objective can be formulated as $X_{t+1}$ must be out of the collision regions of all surrounding objects. To achieve this goal, it requires that:

(7) $$\left(V_{t}+A_{t}\Delta_{t}\right)\cap VO_{t}=\emptyset$$

where $VO_{t}$ is the union of the velocity obstacle region output by Algorithm 1 for the MASS at time *t*.

#### 4.1.2. Sailing Efficiency

Sailing efficiency is required because the MASS desires to reach the destination as early as possible. To achieve this goal, the MASS should select a velocity that has the shortest time expectation to reach the destination. The expected time to reach the target by the adjusted velocity $v_{X}+A_{t}$ can be calculated as follows:

(8) $$T_{E}\left(X,\mathrm{Target}\right)=\frac{∥XT∥}{\langle v_{X}+A_{t},XT\rangle }$$

where XT is the vector from *X* to the target *T*. Its length divided by the MASS’s projected velocity onto XT is the expected time to reach the destination using the adjusted velocity.

#### 4.1.3. Sailing Smoothness

Another requirement is that the MASS should sail as smoothly as possible. It should avoid sharp turning, sharp acceleration, or sharp braking. Therefore, a penalty should be added to sharp velocity changing operations. A weighted combination of turning angle and velocity change is used as the motion smoothness metric.

(9) $$S_{t}=\lambda_{1}\left(\arccos\frac{\langle v_{X}+A_{t},v_{X}\rangle }{∥v_{X}+A_{t}∥∥v_{X}∥}\right)+\lambda_{2}∥A_{t}*\Delta_{t}∥$$

where $\lambda_{1}$ and $\lambda_{2}$ are user preferences for the angle and velocity smoothness.

### 4.2. Combining the Acceleration Constraints

In practice, due to the power and motion constraints of the MASS, the acceleration is restricted by two aspects:

1. The maximum acceleration, i.e., $ACC_{max}$, which is determined by the driving power and breaking force of the ship;
  (10) $$\sqrt{A_{t,x}^{2}+A_{t,y}^{2}}\leq ACC_{\max}$$
2. The ship has a minimum turning radius, which requires that the orthogonal acceleration over the forwarding velocity cannot be too large to avoid ship roll-over during turning.
  (11) $$\frac{∥A_{t}-\frac{\langle v_{X}\cdot A_{t}\rangle }{∥v_{X}∥∥v_{X}∥}v_{x}∥}{∥v_{X}∥}\leq H$$
  where $∥A_{t}-\frac{\langle v_{X}\cdot A_{t}\rangle }{∥v_{X}∥∥v_{X}∥}v_{x}∥$ is the magnitude of acceleration orthogonal to the ship direction; *H* is a threshold.

#### 4.2.1. Discretize the Feasible Acceleration

**Figure 3:**
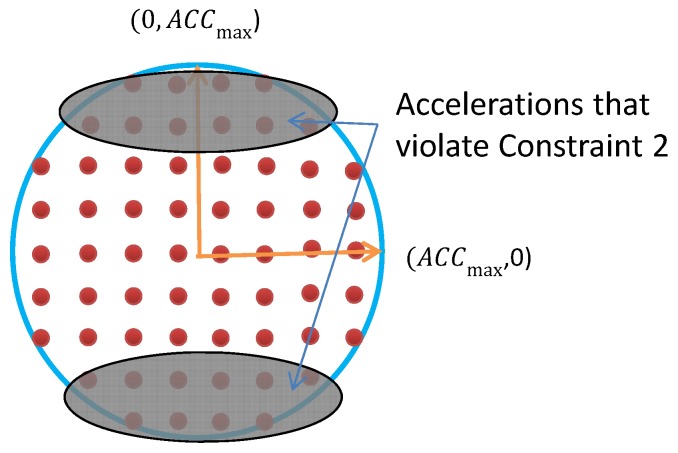
The red points show the discretized possible acceleration vectors at time *t* that satisfy the motion constraints.

Due to the constraints of the maximum acceleration and turning radius, the feasible acceleration will be in a two-dimensional region, as shown in Figure 3. The constraint in (10) restricts the accelerations to be in a circle with radius $ACC_{max}$. The constraint in (11) further excludes a set of accelerations that violate (11). The shaded area excludes the accelerations that may cause the MASS to turn over.

**Figure 4:**
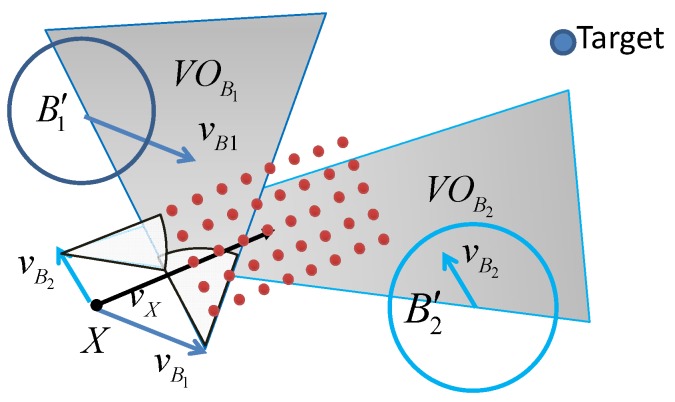
Illustration of the VO model of multiple mobile objects. $B_{1}$ and $B_{2}$ are two mobile objects. The shaded areas show $VO_{B_{1}}$ and $VO_{B_{2}}$, respectively.

In order to optimize the acceleration selection, a reasonable approach is to discretize the continuous feasible region of accelerations to a set of discrete accelerations. The discretized points work as possible candidates for acceleration selection at time *t*. An example of the set of discretized accelerations is show in Figure 3. The discretized acceleration set is denoted by $A_{t}$. Then, from the set of possible accelerations and the velocity update function in (6), a set of possible velocity vectors at time *t* can be obtained, as shown in Figure 4. Let us denote the set of achievable velocity vectors by $V_{t}$. The goal of motion optimization is to select the best velocity update at *t* so that the multiple optimization objectives can be achieved as best as possible.

#### 4.2.2. The One-Interval Optimization Problem

Based on the optimization objectives and the constraints on the accelerations, we can define the one-step optimization problem as follows:

**Problem** **2.** *Given $VO_{t}$ the VOs at t, $X_{t}$ the MASS location at t, T the target location, $V_{t}$ the velocity of the MASS, $\Delta_{t}$ the interval length, $ACC_{max}$ the maximum acceleration, and H the threshold on the turning angle, the one-step optimization problem in one interval can be formulated as:*

(12) $$\begin{array}{l} A_{t}^{*}=\underset{A_{t}}{\arg\;\min}V\left(X_{t}\right)=\left\{\frac{\lambda_{0}∥XT∥}{\langle V_{t}+A_{t},XT\rangle }+\lambda_{1}\left(\arccos\frac{\langle v_{X}+A_{t},V_{t}\rangle }{∥V_{t}+A_{t}∥∥V_{t}∥}\right)+\lambda_{2}∥A_{t}*\Delta_{t}∥\right\}\\ s.t.\left\{\begin{array}{l} \sqrt{A_{t,x}^{2}+A_{t,y}^{2}}\leq ACC_{\max}\\ \frac{∥A_{t}-\frac{\langle V_{t}\cdot A_{t}\rangle }{∥V_{t}∥∥V_{t}∥}V_{t}∥}{∥V_{t}∥}\leq H\\ \left(V_{t}+A_{t}\Delta_{t}\right)\cap VO_{t}=\emptyset \end{array}\right. \end{array}$$

#### 4.2.3. A Greedy Velocity Optimization Algorithm

To solve Problem (2), a greedy algorithm is proposed to optimize the motion plan of the MASS. It contains two steps: (1) The MASS updates the VO model based on surrounding information using Algorithm 1. (2) Then, the MASS adjusts its velocity in a greedy manner to solve Problem (2). This algorithm helps the MASS adjust its velocity to approach the target while avoiding collisions. The details of the Algorithm are given in Algorithm 2. 

**Algorithm 2:** Greedy velocity optimization.

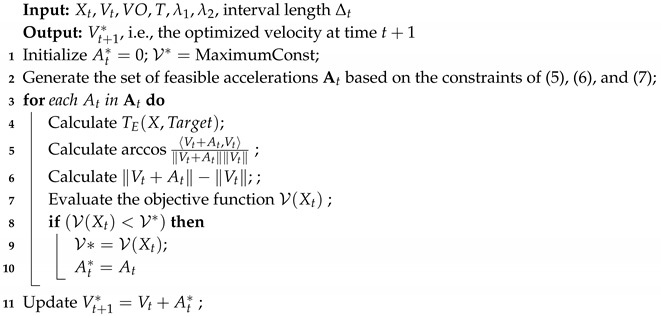



### 4.3. Preventive Control to Avoid Future Collisions

However, the greedy one-step optimization algorithm as shown in Algorithm 2 may fail to give the overall optimal solution to Problem 1. A problem of the greedy optimization is that it has not considered the decision’s impacts on the future steps. An extreme case is that the MASS may have difficulty finding a way out to avoid collisions in the next interval.

#### 4.3.1. Example of Encountering a Collision

**Figure 5:**
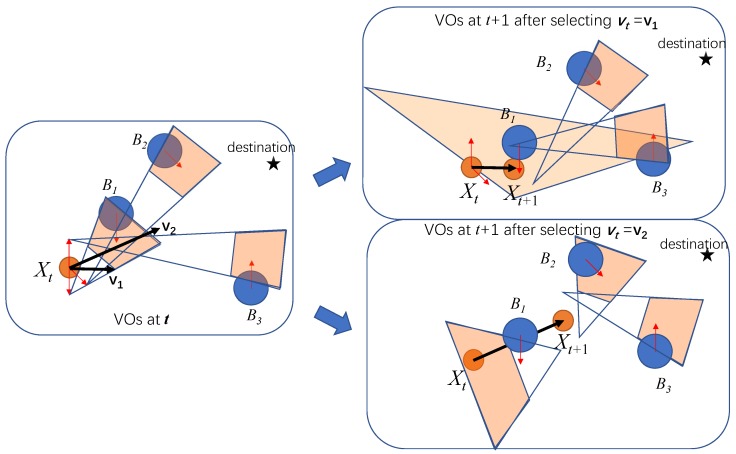
The greedy motion plan in one step may lead to a collision in the next step.

Figure 5 shows an example when the greedy motion in one step leads to a situation where collision is hard to avoid in the next interval. The left sub-figure in Figure 5 shows the situation at time *t*. If the MASS chooses to update its velocity to $v_{1}$, the situation at t+1 is shown in the right-top sub-figure in Figure 5. Since the MASS is very close to another ship $B_{1}$, the VO of $B_{1}$ covers the feasible velocity region of the MASS. Therefore, it becomes hard for the MASS to find a feasible acceleration to avoid a collision with $B_{1}$. The right-bottom sub-figure shows the case when the MASS selects $v_{2}$ at *t*. The MASS arrives at $X_{t+1}$ at t+1, which makes it easy to plan its next motion step.

Obstacles are hard to avoid when the MASS is very close to them. In such a case, its feasible velocity region is highly covered by the VOs of the surrounding ships, and the MASS cannot find a feasible way out to avoid collisions with some ships. In this case, collisions will happen in the future step. An intuitive way for the MASS to avoid such cases is to evaluate the risk of sailing into a region that is closely surrounded by other ships.

#### 4.3.2. Way-Blocking Metric

We propose a way-blocking metric to evaluate this risk. The way-blocking metric is defined by evaluating the proportion of ways around the MASS that are covered by VOs.

**Definition** **3** (Way-blocking metric)**.** *Let C be a circle centered at the location of the MASS and with radius R. Let us define the circumference covered by VOs by CI(VO), then the portion of the circumference of the circle covered by VOs is defined as the way-blocking metric.*

(13) $$B\left(R,P,VO\right)=\frac{CI(VO)}{2\pi R}$$

To reduce the probability of falling into local optima, the MASS can add the consideration of the way-blocking metric to the speed scheduling metric.

(14) $$A_{t}^{*}=\underset{A_{t}\in A_{t}}{\mathrm{argmin}}V\left(X_{t}\right)=\left\{\begin{array}{c} T_{E}\left(X,\mathrm{Target}\right)\\ +\lambda_{1}\left(\arccos\frac{\langle V_{t}+A_{t},V_{t}\rangle }{∥V_{t}+A_{t}∥∥V_{t}∥}\right)\\ +\lambda_{2}{\left(∥V_{t}+A_{t}∥-∥V_{t}∥\right)}^{2}\\ +\lambda_{3}B\left(R,P,VO,A_{t}\right) \end{array}\right\}$$

where $B(R,P,VO,A_{t})$ evaluates the way-blocking metric at the next time interval. Therefore, we can revise the greedy velocity optimization algorithm by replacing the cost function by (14), which is called a greedy-risk algorithm.

## 5. Multiple Interval Optimization by Dynamic Programming

The example in Figure 5 provides hints about the multiple interval optimization by dynamic programming. By selecting $A_{t}$ at time *t*, the MASS’s location and velocity at the next interval can be calculated as $V_{t+1}$ and $X_{t+1}$. If we assume the other ships are sailing in the same direction at the same velocity in the following intervals, the VOs for the MASS at time t+1 can be predicted as:

(15) $$VO_{t+1}=F\left(X_{t},V_{t+1}\right)$$

### 5.1. The Recursive Cost Function

Based on $VO_{t+1}$, the MASS can optimize its motion at t+1 to minimize its cost function to reach the destination, denoted by $V(X_{t+1})$, while satisfying all the motion and collision avoidance constraints. Therefore, we can set up a recursive cost function. If the MASS is at the target, the cost to reach the target is zero. Then, the cost at $X_{t}$ is the minimum sum cost of $V\left(X_{t+1}\right)+A\left(X_{t},V_{t+1}\right)$, where $A(X_{t},V_{t+1})$ is the one-step motion cost for moving from $X_{t}$ to $X_{t+1}$. The constraint is $X_{t}+V_{t+1}\Delta_{t}=X_{t+1}$. Therefore, the recursive cost function can be set up as:

(16) $$\left\{\begin{array}{l} V(T)=0\\ V\left(X_{t}\right)=\min_{X_{t}+V_{t+1}\Delta_{t}=X_{t+1}}\left\{\eta V\left(X_{t+1}\right)+A\left(X_{t},X_{t+1}\right)\right\} \end{array}\right.$$

where 0<η<1 is a discounting parameter. The one-step motion cost is the time cost of moving from $X_{t}$ to $X_{t+1}$ using velocity $V_{t+1}$ plus the smoothness cost.

(17) $$A\left(X_{t},X_{t+1}\right)=\left\{\frac{\lambda_{0}∥X_{t}-X_{t+1}∥}{\langle V_{t}+A_{t},XT\rangle }+\lambda_{1}\left(\arccos\frac{\langle V_{t}+A_{t},V_{t}\rangle }{∥V_{t}+A_{t}∥∥V_{t}∥}\right)+\lambda_{2}∥A_{t}*\Delta_{t}∥\right\}$$

### 5.2. The Multiple Interval Motion Optimization Algorithm

Based on the recursive cost function, the multiple interval motion optimization algorithm is designed. It is based on the fact that: given $X_{t}$ and $V_{t+1}$, $X_{t+1}$ can be inferred, and the VO model can be inferred by assuming the other ships are sailing at a constant speed. However, the VOs are related to the MASS’s position. Therefore, the VOs for different $X_{t+1}$ should be calculated respectively. The problem for the dynamic programming approach is that the computational complexity to infer VOs for all possible multiple interval scenarios is high. There are two ways to reduce the computational cost:

1. Discretize the map into grids with a coarser size.
2. Only foresee a limited number of future intervals.

The first way can reduce the number of discretized MASS states, and the second method can reduce the looking forward steps. The second constraint is realized by defining a common cost surface. When the MASS is on any grid of the surface, its future costs to the target are set the same. Therefore, the costs of early intervals can be defined based on the cost of the surface. The dynamic programming-based multiple interval motion planning algorithm is designed. The dynamic programming method has two steps: (1) the graph for iterative motion cost calculation is established first on the discrete map based on the MASS’s motion constraint and the VOs calculated at each interval; (2) the cost of each path is calculated, and the optimal motion path is given.

#### 5.2.1. Construct the Cost Graph

**Figure 6:**
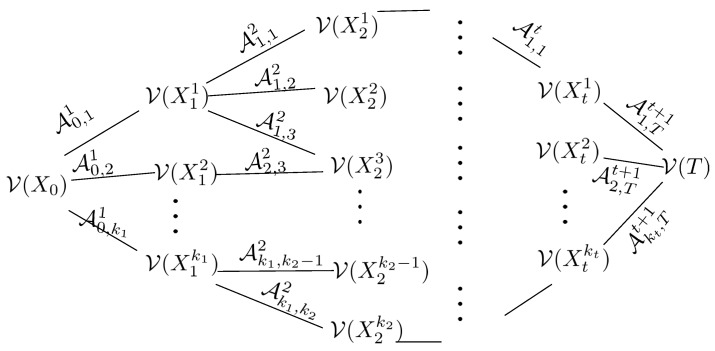
An illustration of the multiple interval dynamic programming approach for motion planning.

The cost graph is constructed based on the motion constraints and the VOs of the MASS at each time interval on the discrete map. An illustration is shown in Figure 6. The initial location of the MASS is $X_{0}$. The VOs are calculated based on $X_{0}$, the locations of other ships, and their velocities. Suppose $X_{1}^{1}$ to $X_{1}^{k_{1}}$ are the MASS’s reachable grids without collision after the first interval. $A_{0,i}^{1}$ denotes the one-step motion cost from $X_{0}$ to $X_{1}^{i}$, which is calculated by (17), and $V(X_{1}^{i})$ denotes the cost function of location $X_{1}^{i}$. Then, from each $X_{1}^{i}$, the VOs can be calculated based on the predicted locations of other ships and their velocities. The cost graph can be expanded to the second interval. The process repeats until the MASS is at the target *T*.

#### 5.2.2. Find the Optimal Motion Path

Since V(T)=0, when the cost graph is constructed, the costs of middle layer nodes can be calculated based on (16). Then, the optimal motion plan can be found from $X_{0}$ to *T*, which is the path that generates the cost for $V(X_{0})$. The detail of the algorithm is given in Algorithm 3.

**Algorithm 3:** Multiple interval velocity optimization.

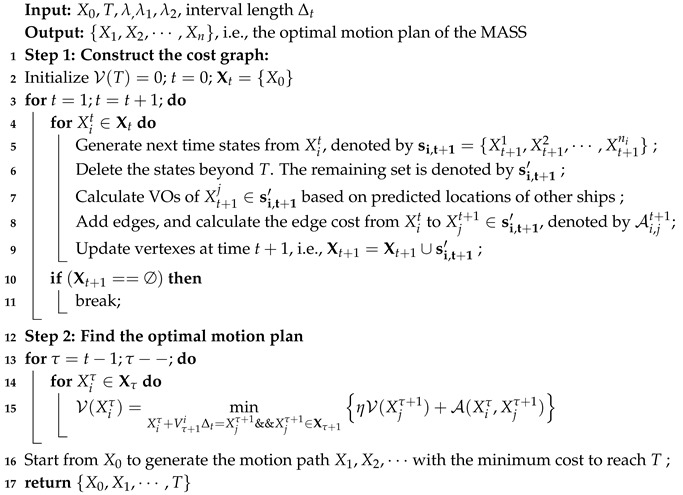



The first step to generate the cost graph starts from $X_{0}$ and then expands the next states at interval t+1 until beyond the destination. The edges and one-step costs on the edges are generated by (17). The second step evaluates the minimum cost of each node in the graph. The optimal motion path is the path that derives the minimum cost for $X_{0}$.

## 6. Simulation and Evaluations

### 6.1. Simulation Settings

Simulations were conducted to evaluate the effectiveness of the proposed velocity optimization schemes for MASS. The simulation was conducted in MATLAB 2018. We simulated the scenario when an MASS was sailing in an L∗W region from (0 km, 0 km) to (1000 km, 1000 km) when there were *K* conventional ships moving. We assume the MASS can detect the shape, the location, and the velocity of the conventional ships. This simulates the case when the locations and velocities of other ships are provided by AIS. We simulated three algorithms:

1. greedy: the greedy one-step velocity optimization;
2. greedy + risk: the greedy one-step velocity optimization with consideration of collision risk;
3. DP: the dynamic programming-based multiple interval motion planning;

### 6.2. Performance Metrics

Sailing efficiency, in terms of the time to reach the destination, is an important performance metric of the motion control algorithms. We evaluated the difference of sailing efficiency of the greedy algorithm using metric function (10), denoted as greedy, and metric function (11) denoted as greedy + risk, respectively. The sensing noises were considered by enlarging the ship radius *r*.

Collision avoidance is also an important performance metric. The VO-based greedy velocity scheduling algorithm may fall into local optima when it reaches a position that is surrounded by moving ships. In this case, collisions will happen, because the ship cannot find a feasible way to avoid collisions. The greedy + risk algorithm avoids collisions by considering the risks of falling into local optima. Several parameters may impact the probability of collisions happening.

1. the density of surrounding ships, denoted by *d*;
2. the velocities of other ships, denoted by $v_{s}$;

We evaluate the collision avoidance performances of greedy, greedy + risk, and dynamic programming by varying the various parameters.

### 6.3. Simulation Results

#### 6.3.1. Snapshots of Simulations

**Figure 7:**
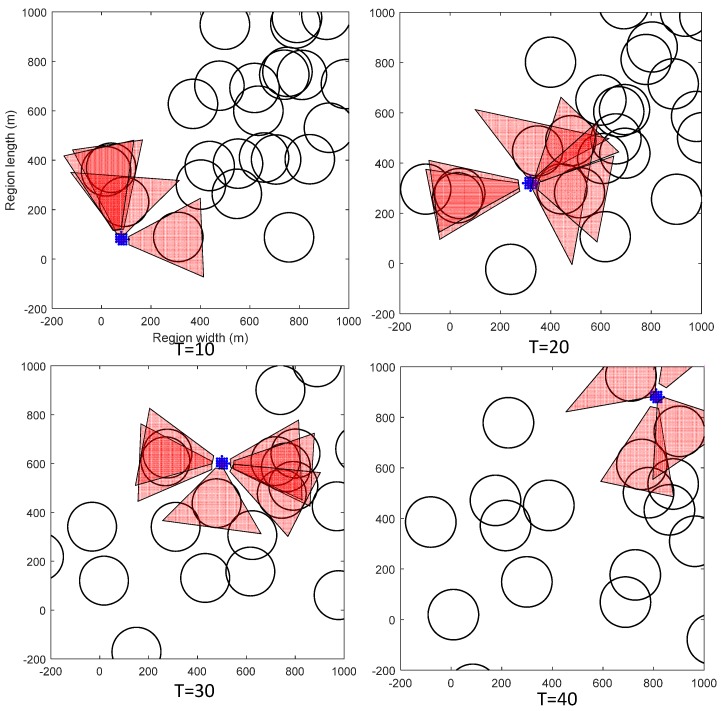
Four snapshots in the simulation when the MASS started from (0, 0) with destination (1000, 1000). The black circles are collision regions with other ships that should be avoided. The red cones show the VOs.

Figure 7 shows four snapshots for one experiment in the simulation. The MASS is represented by the small red circle. The VOs are represented by the cones. The MASS starts from (0, 0) and finally reaches (1000, 1000) using the greedy speed scheduling metric. The unit is kilometers. We can see that the MASS can successfully avoid collisions with other ships, even when it is closely surrounded by other mobile ships.

Collisions happen only when the way-blocking metric reaches one, such that all ways of escape have been blocked by other ships. The density of surrounding ships, the velocity of other ships, and the tolerable maximum way-blocking metric all affect the probability of collisions happening. We evaluated the impacts of these parameters on the probability of collisions happening. Note that in the ideal case when the other ships are sailing at a constant velocity, the dynamic programming method can avoid collisions since the optimal trajectory is selected under the collision avoidance condition. However, the surrounding ships may change their sailing patterns. In the simulation, we added 1% noise to the surrounding ships’ velocities and directions in each time interval. This noise may cause collisions of the MASS even in the DP method. Therefore, we also evaluated the collision probabilities of MASS in the DP method. Under each parameter setting, the experiments were run 100 times to evaluate the probability of collisions happening. In these experiments, the maximum velocity of the MASS was 100 km/h, and the maximum acceleration was also 100 km/h${}^{2}$.

#### 6.3.2. Collision Probability of Greedy Algorithms vs. Ship Density in the Region

**Figure 8:**
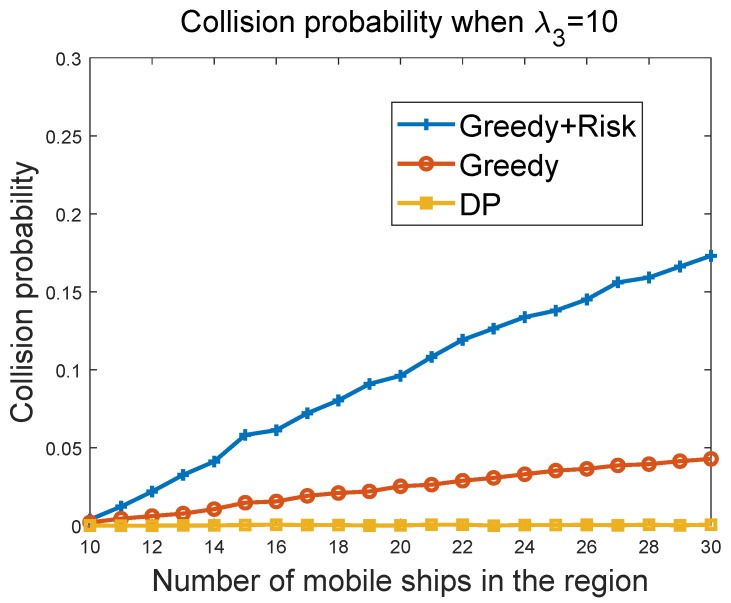
Collision probability vs. ship density when $\lambda_{3}=10$.

**Figure 9:**
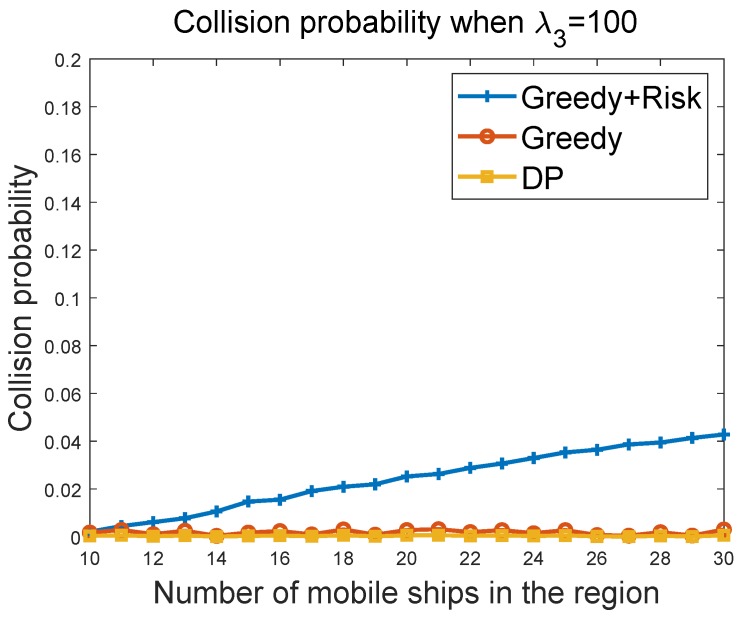
Collision probability vs. ship density when $\lambda_{3}=100$.

Figure 8 and Figure 9 show the collision probability of the MASS with other ships as a function of the ship density in the region. Note that collisions happen when the MASS cannot figure out a way to avoid collisions with other ships at some time instant. We compared the performance of the greedy algorithm and the greedy + risk algorithm when $\lambda_{3}=10$ and $\lambda_{3}=100$, respectively. The greedy + risk algorithm showed better performance for collision avoidance when higher weights were assigned to the way-blocking metric in the greedy algorithms. The DP algorithm provided the lowest collision probabilities under different parameter settings.

#### 6.3.3. Collision Probability of Greedy Algorithms vs. Maximum Velocity of Surrounding Ships

**Figure 10:**
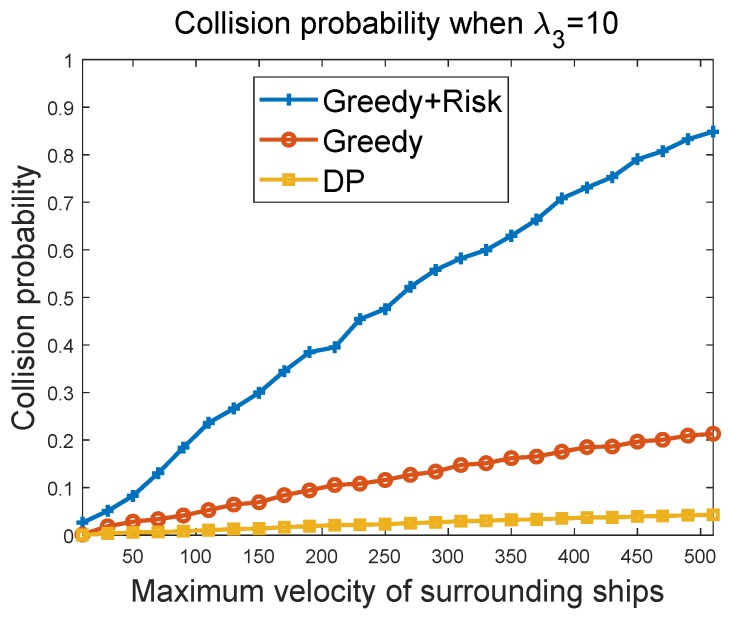
Collision probability vs. maximum velocity of other ships when $\lambda_{3}=10$.

**Figure 11:**
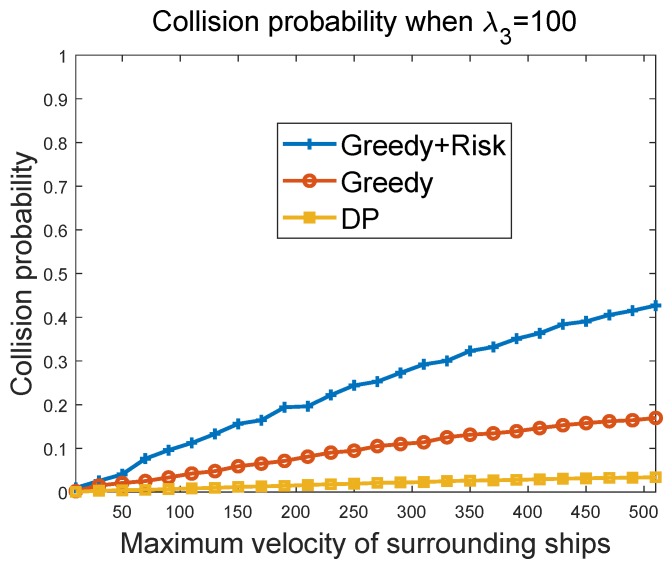
Collision probability vs. maximum velocity of other ships when $\lambda_{3}=100$.

Figure 10 and Figure 11 show the collision probability of the MASS as a function of the maximum velocity of other ships in the region. The collision happens when the MASS cannot figure out a way to avoid collisions with other ship at some time instant. The performances of the greedy algorithm and the greedy + risk algorithm when $\lambda_{3}=10$ and $\lambda_{3}=100$ were compared. It can be seen that the greedy + risk algorithm showed better performance for collision avoidance when higher weights were assigned to the way-blocking metric. However, in these experiments, we found that when the surrounding ships had a high velocity, there were high probabilities of collisions, even if the weights to the way-blocking metric were quite high. In these experiments, we set the number of surrounding ships to 20. The DP algorithm provided low collision probabilities, but it achieved these by paying a higher cost in waiting to avoid the obstacles.

#### 6.3.4. Time to Reach Destination vs. the Number of Ships in the Region

**Figure 12:**
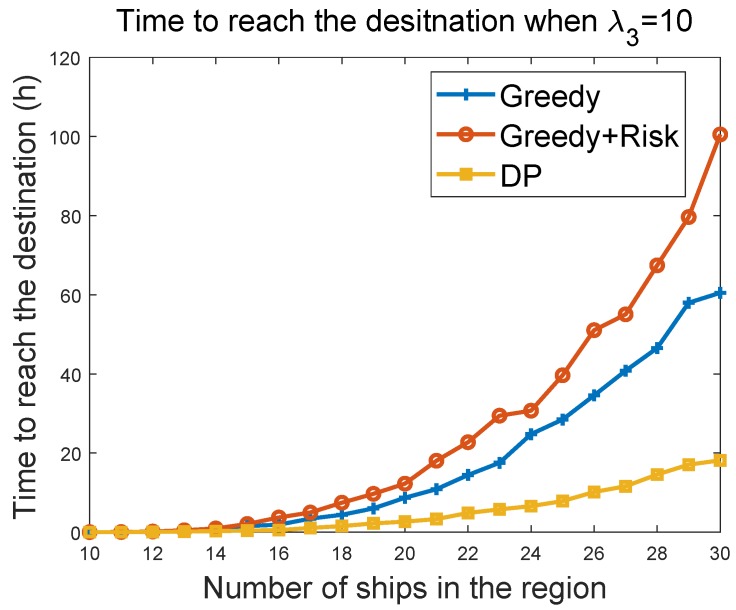
Time to destination vs. density of ships when $\lambda_{3}=10$.

**Figure 13:**
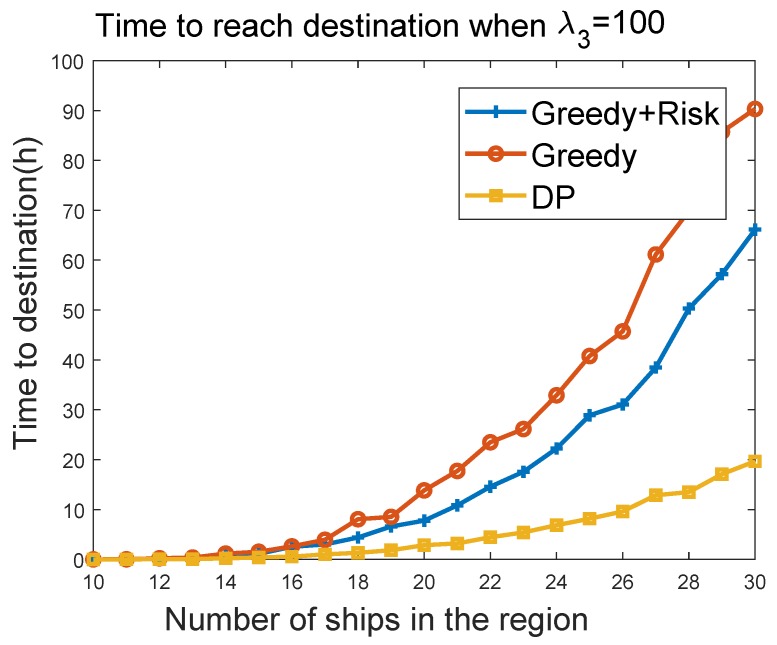
Time to destination vs. density of ships when $\lambda_{3}=100$.

We further evaluated the sailing efficiency performances of greedy, greedy + risk, and the DP algorithms. By setting the maximum velocity of the MASS to 100 and the maximum acceleration to 100, we evaluated the time to reach a target at (5000 km, 5000 km), when the MASS started from (0, 0). The target was reached when the MASS was within a 100-m distance of the target. The sailing efficiency was evaluated by varying the density of surrounding ships. The surrounding ships had the maximum velocity of 100 km/h in the experiments. The range of 0–500 in the *x*-axis of Figure 12 maps to 0–100 m/h in the velocity of the ships. Figure 12 and Figure 13 show the evaluation result of the time to reach the destination as a function of number of ships in the region. It can be seen that the greedy algorithm had better sailing efficiency, such that the MASS reached the target earlier than that of greedy + risk algorithm. When $\lambda_{3}$ was changed from 10 to 100, the sailing efficiency of greedy + risk was reduced a little bit. DP provided overall the best sailing efficiency in the compared algorithms. DP was also less impacted by the parameter $\lambda_{3}$. Note that in all of these evaluations, we counted only the trip in which the MASS successfully reached the destination without colliding with other ships.

## 7. Conclusions

This paper investigates the motion control problem of Maritime Autonomous Surface Ships (MASS) for not only collision avoidance, but also for speed optimization and sailing smoothness. By utilizing the rich information of the locations and velocities of surrounding ships and remote ships, the VO model is exploited for collision avoidance. A multiple objective optimization problem model is set up, and three algorithms are proposed to address the motion planning optimization problem: (1) a greedy motion-planning algorithm, (2) a greedy algorithm with consideration of future collision risk, and (3) a dynamic programing-based multiple interval motion-optimization algorithm. The dynamic programming method utilizes the remote information provided by AIS, which was shown to provide better sailing safety and efficiency than the two greedy algorithms. However, the DP algorithm needs accurate motion information of surrounding ships. If there are vessels whose motions are not captured by AIS, the MASS still needs the interval-based greedy method to avoid local collisions. We will study the joint work of DP and the locally-greedy algorithm in future work.
